# A policy implementation study of earmarked taxes for mental health services: study protocol

**DOI:** 10.1186/s43058-023-00408-4

**Published:** 2023-03-31

**Authors:** Jonathan Purtle, Nicole A. Stadnick, Megan Wynecoop, Eric J. Bruns, Margaret E. Crane, Gregory Aarons

**Affiliations:** 1 Department of Public Health Policy & Management, Global Center for Implementation Science, New York University School of Global Public Health, 708, Broadway, New York, NY 10003 USA; 2 Department of Psychiatry, Dissemination and Implementation Science Center, Altman Clinical and Translational Research Institute, University of California San Diego, 9500 Gilman Drive, La Jolla, CA 92093 USA; 3 Department of Psychiatry and Behavioral Sciences, University of Washington School of Medicine, 6200 NE 74Th St, Building 29, Suite 110, Seattle, WA 98115 USA; 4 Department of Psychology, Temple University, Weiss Hall, 1701 N 13Th St, Philadelphia, PA 19122 USA; 5 Department of Psychiatry, New York Presbyterian-Weill Cornell Medicine, 425 E 61St St, New York, NY 10065 USA

**Keywords:** Policy implementation, Financing, Taxes, Mental health

## Abstract

**Background:**

Insufficient funding is frequently identified as a critical barrier to the implementation and sustainment of evidence-based practices (EBPs). Thus, increasing access to funding is recognized as an implementation strategy. Policies that create earmarked taxes—defined as taxes for which revenue can only be spent on specific activities—are an increasingly common mental health financing strategy that could improve the reach of EBPs. This project’s specific aims are to (1) identify all jurisdictions in the USA that have implemented earmarked taxes for mental health and catalogue information about tax design; (2) characterize experiences implementing earmarked taxes among local (e.g., county, city) mental health agency leaders and other government and community organization officials and assess their perceptions of the acceptability and feasibility of different types of policy implementation strategies; and (3) develop a framework to guide effect earmarked tax designs, inform the selection of implementation strategies, and disseminate the framework to policy audiences.

**Methods:**

The project uses the Exploration, Preparation, Implementation, Sustainment (EPIS) framework to inform data collection about the determinants and processes of tax implementation and Leeman’s typology of implementation strategies to examine the acceptability and feasibility strategies which could support earmarked tax policy implementation. A legal mapping will be conducted to achieve aim 1. To achieve aim 2, a survey will be conducted of 300 local mental health agency leaders and other government and community organization officials involved with the implementation of earmarked taxes for mental health. The survey will be followed by approximately 50 interviews with these officials. To achieve aim 3, quantitative and qualitative data will be integrated through a systematic framework development and dissemination process.

**Discussion:**

This exploratory policy implementation process study will build the evidence base for outer-context implementation determinants and strategies by focusing on policies that earmarked taxes for mental health services.

**Supplementary Information:**

The online version contains supplementary material available at 10.1186/s43058-023-00408-4.

Contributions to the literature

- Policy-focused research remains underdeveloped in the field of implementation science and there are a limited number of published study protocols focused on policy issues.
- This protocol provides an example of how a specific policy—those which earmark tax revenue for mental health services—can function as the focus of an implementation science study.
- Despite the increasing use of earmarked taxes for mental healthcare, there is scant research on the extensiveness of these policies or the nature of their implementation.
- This protocol illustrates how existing implementation science frameworks, constructs, and survey items can be adapted for policy-focused implementation research.
- This protocol details how legal mapping research methods can be integrated into a policy-focused implementation science study.

## Background

Insufficient funding is frequently identified as a barrier to the implementation and sustainment of evidence-based practices (EBPs) [1–4]. As such, increasing access to funding is recognized as a promising implementation strategy [5].Policies that create earmarked taxes—defined as taxes for which revenue can only be spent on specific activities—are an increasingly common financing strategy that hold promise for improving the reach of EBPs [6–8]. However, little is known about how the design and implementation of earmarked tax policies might be optimized to reflect local contexts and also ensure that revenue is allocated for practices that are effective. This exploratory, mixed methods (QUANT➔ QUAL), policy implementation process study [9] will expand the evidence base for outer-context implementation determinants and strategies by focusing on the implementation of policies that earmark taxes for mental health services. The project uses the Exploration, Preparation, Implementation, Sustainment (EPIS) framework [10] to inform data collection about the determinants and processes of tax implementation and Leeman et al.’s typology of implementation [11] to examine the acceptability and feasibility of strategies that could support earmarked tax policy implementation. The project will contribute to a growing body of empirical research about health policy implementation in the USA.

### Study aims

The study has three aims.

- *Aim 1: Identify all jurisdictions in the USA that have implemented earmarked taxes for mental health services and catalogue information about tax design.* Using recommended practices for legal mapping studies [12–14], key informant interviews and legal mapping will be conducted to identify policies that create earmarked taxes for mental health services and catalogue information on tax design.
- *Aim 2: Characterize local government and community organization leaders’ experiences implementing earmarked taxes, understand the determinants of decisions about tax-funded programs, and assess the acceptability and feasibility of different types of implementation strategies*. A web-based survey will be conducted of 300 local (e.g., county, city) mental health agency leaders and other government and community organization officials involved with tax implementation. Approximately 50 semi-structured interviews will then be conducted with these leaders and officials in purposively selected counties.
- *Aim 3: Develop a conceptual policy implementation framework to guide effective earmarked tax designs, inform the selection of implementation strategies to increase the taxes’ reach of EBPs, and disseminate the framework to relevant policy audiences.* An established, systematic process [15] will be used to integrate quantitative survey and qualitative interview data and develop a framework focused on the design and implementation of earmarked taxes for mental health. Then, using empirically-informed dissemination practices [16–21], we will disseminate the framework to policymakers and implementers in jurisdictions that have implemented the taxes.

### Earmarked taxes as health policy strategy

Earmarked taxes are those placed on specific goods, services, or income for which revenue is dedicated to a specific purpose [6, 8, 22, 23]. Earmarked taxes have become increasingly common at state and local levels in the USA across policy areas for which the public strongly supports government intervention (e.g., transportation, education) [24]. The increasing popularity of earmarked taxes likely stems, at least in part, from decreases in public support for general tax increases and declines in trust of government (especially at the federal level) [25, 26]. Earmarked taxes often enjoy relatively strong public support because they guarantee that revenue will be allocated for specific issues of public concern [24, 27], as opposed to being allocated at the discretion of government officials who are increasingly perceived as untrustworthy [25, 26]. Although these taxes have the potential to produce a net increase in spending on an issue by creating a new funding stream [28], this may not occur due to supplantation—the process through which spending on an issue is reduced from the general fund because the issue already has a separate and dedicated (i.e., earmarked) revenue source [29–32].

In the area of health, earmarked taxes have typically been applied on goods and services that produce harms to public health [33, 34]. In these cases, earmarked taxes have the dual goal of reducing consumption of the good or service *and* generating revenue for investments in public health. Widely studied examples of earmarked taxes in the area of health include excise taxes on sugar sweetened beverages [35, 36], indoor tanning [37], alcohol [38], and tobacco [39] with revenue earmarked for public health programs that address these issues.

### Earmarked taxes for mental health services

In the area of mental health, earmarked taxes have been adopted with the goal of increasing funding for mental health services—which have historically been funded less generously than physical health services in the USA [40–44]. As described in a 2019 commentary [6], two US states—California and Washington—adopted high profile earmarked tax policies for mental health services in 2005. These policies, however, differ dramatically in terms of tax design and oversight.

In California, the Mental Health Services Act increased the income tax rate by one percentage point for households with annual income exceeding $1 million. Revenue is collected by the state and then allocated to all counties in the state using a formula that accounts for population size and other characteristics [45]. The California Mental Health Oversight and Accountability Commission oversees highly structured spending and reporting requirements. Studies have assessed the impact of the California tax and tax-funded programs on effectiveness outcomes (e.g., suicide death, mental illness stigma) [46–48] and implementation outcomes related to the adoption and sustainment of tax-funded services [49, 50]. Prior research has not, however, focused on the processes of tax implementation in California, perceptions of tax design, or perceptions of strategies that could improve implementation.

In contrast, Washington state law E2SSB-5763 provided counties with the ability to raise their sales tax rate by 0.1% percentage point, via referendum, to increase funding for mental health services. As of 2022, 28 of the 39 counties in the state had adopted the tax, with adoption occurring gradually across the state since 2005. Counties that adopt the tax are required to establish a therapeutic substance use disorder court [51] and report information about the amount of revenue generated to Washington State Department of Revenue. However, in contrast to California, counties monitor spending without structured state oversight and have fairly broad discretion over the specific services which are funded. Little research has investigated the implementation or effects of these taxes in Washington counties.

As the 2019 commentary also described, local jurisdictions in states such as Illinois, Colorado, and Missouri have also adopted policies that earmark taxes for mental health services. However, details about these taxes—or others than may exist across the USA—have not been systematically collected. It is plausible that additional jurisdictions will adopt policies earmarking taxes for mental health services as public concern about mental health is extremely high in the USA [52]. Furthermore, many US adults are willing to pay higher taxes to improve mental health services systems [53–55]. One 2017 survey found that 42% of respondents were willing to pay an additional $50 annually to improve the mental health service system [53]. A separate survey conducted the same year found that 58% were willing to pay an additional $50 for social services for people with serious mental illness [54]. A 2018 discrete choice experiment found that support for increased spending on mental health was higher than for other health and social issues [55]. It is within this context that policies that earmark taxes for mental health services have emerged as a financing strategies in the USA.

### Policy is a growing area of implementation science research

Although policy implementation has been an area of focus in fields such as public administration and management research and political science since at least the mid-twentieth century [56–61], public policy has historically been understudied in the contemporary enterprise of implementation science in health [62]. However, interest in the area is rapidly growing. Implementation science researchers have recently published calls for a greater emphasis on policy in the field [9, 63–68], and reviews have cataloged measures and strategies for policy-focused D&I research [69–72]. Trials have tested policymaker focused dissemination strategies [73–80], and conceptual frameworks have been developed and refined for policy-focused implementation science [81, 82]. Definitions of implementation science concepts (e.g., implementation strategies) have been adapted for policy-focused work [83, 84], studies have evaluated the effects of policies on implementation outcomes [85, 86], and protocols have detailed studies that focus on policy dissemination and implementation [87–90]. This study contributes to this growing area of research in policy-focused implementation science.

## Methods

**Fig. 1:**
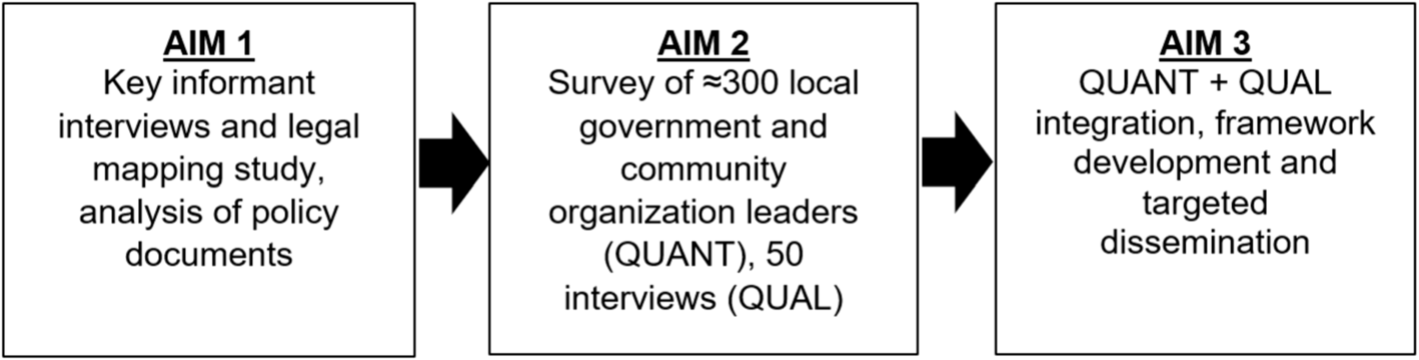
Study process

This exploratory project uses a sequential mixed method (QUANT➔ QUAL) design and progresses across three phases (Fig. 1).

### Conceptual frameworks

**Fig. 2:**
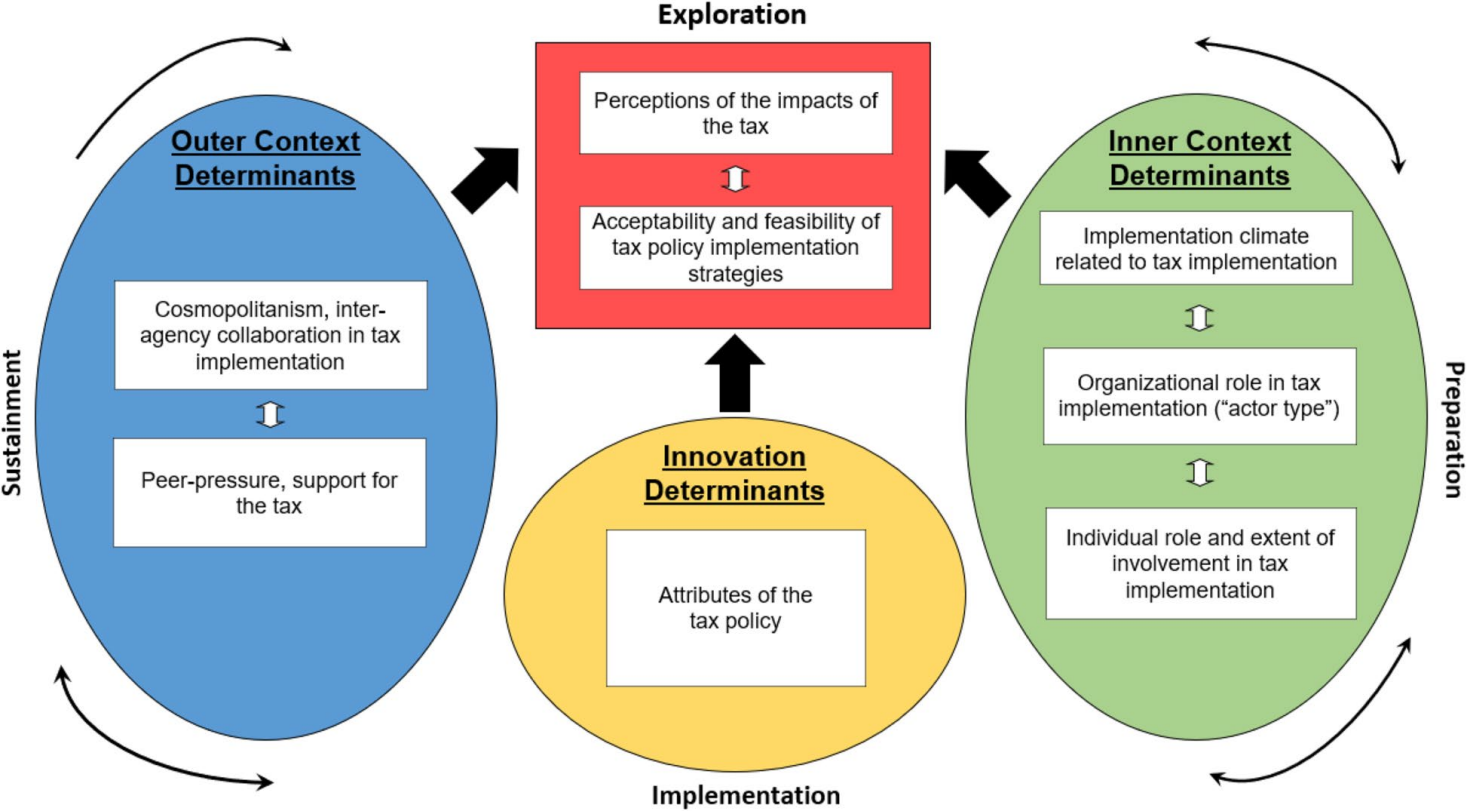
Study conceptual framework

The project is guided by two D&I frameworks—the EPIS framework [10] and Leeman et al.’s implementation strategy typology [11]. EPIS is both a process and determinant framework that was developed for implementation research in public sector settings [91]. Acknowledging the multilevel and often non-linear nature of implementation research and practice, EPIS delineates key outer context, inner context, and innovation determinants that may influence implementation across the phases of exploration, preparation, implementation, and sustainment. EPIS will be used to inform assessment of local government and community organization leaders’ experiences implementing earmarked taxes and perceptions of factors that influence implementation. More specifically, as shown in Fig. 2, EPIS informs the selection of variables in the domains of outer context determinants (cosmopolitanism and peer pressure), inner context determinants (implementation climate, role of organization in tax implementation, role of the individual within their organization), and innovations determinants (perceived attributes of the tax, drawing from Rogers’s “attributes of innovations”[92]). EPIS also will be used to guide data interpretation regarding how these determinants are associated with perceptions of the impact of the tax and the acceptability and feasibility of strategies that could be used to help ensure that the tax increases the reach of EBPs. More details about these constructs and their measurement are provided below.

Leeman et al. [11] developed a five-domain classification system for implementation strategies from Powell et al.’s Expert Recommendations for Implementing Change compilation [5]. This typology will be used to develop a survey about the acceptability and feasibility of implementation strategies that could help earmarked taxes increase reach of EBPs. The five domains of the typology are as follows: [1] dissemination strategies (e.g., communicating information about EBPs), [2] implementation process strategies (e.g., adapting EBPs for context), [3] integration strategies (e.g., revising professional roles to support EBP delivery), [4] capacity-building strategies (e.g., technical assistance to support EBP delivery), and [5] scale-up strategies (e.g., training providers in EBPs). As noted below, all survey items assessing the acceptability and feasibility of these strategies will be explicitly anchored to the implementation of earmarked taxes for mental health services [11].

### Aim 1 methods

Aim 1 methods consist of a legal mapping study to identify all jurisdictions in the USA that have implemented earmarked taxes for mental health services and cataloging information on tax design. Aim 1 methods reflect recommended practices for legal mapping studies [12–14].

### Key informant interviews with subject matter experts

Approximately 12 key informant semi-structured interviews will first be conducted with policy directors of mental health professional associations (e.g., American Psychiatric Association), advocacy organizations (e.g., Mental Health America), and experts on tax law (e.g., the Tax Foundation). The purpose of the interviews will be threefold: [1] to identify jurisdictions that have implemented taxes, [2] to inform the search strings used to identify additional jurisdictions through legal databases and other sources, and [3] to inform development of the policy coding instrument. All interviews will be telephone or Zoom-based, recorded, transcribed, and analyzed using rapid, directed content analysis [93].

### Tax policy identification, coding, and analysis

After finalizing search strings, we will search legal databases (e.g., HeinOnline, Cheetah tax repository), reports, and a range of municipal data sources to identify the text of policies that earmark taxes for mental health services. We will extract information on five key attributes of each tax: jurisdiction, year enacted, tax type (e.g., income, property, sales), tax rate, and amount of revenue generated annually. 2020 US Decennial Census estimates of population size within each jurisdiction will then be used to calculate estimates of annual revenue per capita. Property tax “millage rates,” which are expressed dollars per $1000 property valuation, will be converted to percentages to facilitate consistent interpretation with sales and income tax rates. Descriptive statistics will characterize the attributes of the taxes across jurisdictions and brief narrative case studies will also be used to describe the taxes, their history, and synthesize any existing research related to tax evaluation.

### Aim 2 methods

Aim 2 methods consist of a quantitative, web-based survey of 300 local (e.g., county, city) mental health agency leaders and other government and community organization officials involved with tax implementation in the jurisdictions that have implemented earmarked taxes for mental health, followed by a target sample of 50 semi-structured qualitative interviews in purposively selected jurisdictions.

### Survey sample, recruitment, and data collection

In each jurisdiction identified as having an earmarked tax for mental health, we will identify local mental health agency leaders and other government and community organization officials who appear—based on their title as it relates to the tax—to be involved with tax oversight, decision making, implementation, and/or service deliver. We will identify these individuals through Internet searches, contact databases maintained by practice partners (e.g., county mental health association with states), and databases of state local mental health officials compiled by the research team in prior research [94–96].

Everyone in the sample frame will be e-mailed up to eight times over an eight-week period with a unique link to complete the web-based survey in Qualtrics. All recruitment materials will the personalized to include the respondent’s name and title and will concisely describe the earmarked tax in their jurisdiction. Telephone follow up will be conducted to ensure that the e-mails were received and to answer any questions about the survey. Survey recruitment will occur in two waves. The first wave will recruit individuals in the original sampling frame, and the second wave will recruit individuals identified through recommendations obtained from wave 1 surveys and individuals from any new jurisdictions identified after wave 1 survey recruitment has begun. If necessary to meet recruitment milestones, we will also circulate an open survey link via our practice partners (e.g., state and county mental health professional associations).

### Survey measures

Table 1 shows the domains of the web-based survey (survey as Supplemental File 1). The survey is designed with the goal of having a low response burden (i.e., take < 15 min to complete) while covering five conceptual areas—spanning domains of the EPIS framework and Leeman et al.’s typology of implementation strategies. All items will be anchored in reference to the earmarked tax for mental health in the respondent’s jurisdiction. Prior to fielding, the survey instrument will be piloted with five people who have been involved with the implementation of policies that earmark tax revenue for mental health services.

**Table 1:** Survey domains

Core domain	Sub-domain	Example item
Perceptions of the impacts of the earmarked tax	n/a	“Rate the extent to which you agree with the following statements about the impacts of the earmarked tax for mental health in your jurisdiction… The tax increases funding for direct mental health/social services”
Innovation determinants	Attributes of the tax	“Rate the extent to which agree with the following statements about the characteristics of the earmarked tax for mental health in your jurisdiction… The rules related to how revenue from the tax can be spent can be easily changed to address emergent needs”
Inner context determinants	Organizational role in tax implementation (“actor type”)	“Which of one the following categories most accurately describes your organization’s role within the context of implementing the earmarked tax… Providing direct mental health and social services with tax revenue”
	Individual role and extent of involvement in tax implementation	“Rate the extent to which have you have personally been involved with the following activities… Making decisions about what services to fund with tax revenue”
	Implementation climate related to tax implementation	“Indicate the extent to which you agree with each of the statements below about your organization… One of this organization’s main goals is to use evidence-based practices effectively with earmarked tax revenue”
Outer context determinants	Cosmopolitanism, inter-agency collaboration in tax implementation	“Indicate how often you collaborate with each of the following on issues related to implementation of the earmarked tax…. Local child welfare agency/child protective services”
	Peer-pressure, support for the tax	“Rate the extent to which you agree that there is strong support for the tax among… The general public in my jurisdiction”
Acceptability and feasibility of policy implementation strategies	Acceptability of strategy	“Dissemination strategies: These strategies entail your organization communicating information to mental health service organizations to increase leaders and providers knowledge and improve their attitudes about evidence-based practices that can be funded with earmarked mental health tax revenue… Dissemination strategies meet my approval”
	Feasibility of strategy	“Capacity-building strategies: These strategies entail your organization increasing the capacity of mental health service organizations to select and integrate evidence-based practices funded by earmarked mental health tax revenue and evaluate their impacts (e.g., by enhancing the motivation and self-efficacy of leadership and direct service providers) Capacity-building strategies seem easy to use”

### Perceptions of the impacts of the earmarked tax

These perceptions will be assessed by 10 items that ask respondents to indicate the extent to which they agree with statements about positive and negative impacts of the tax. These items will be informed by aim 1 key informant interviews and literature on the potential benefits and drawbacks of earmarked taxes [6, 8, 22–32]. Perceptions will be assessed on Likert scales and those focused on negative impacts will be reverse coded. If internal consistency is reasonable (i.e., *α* ≥ 0.70), these items will be summed to create an aggregate score of the perceived benefits of the earmarked tax for mental health within the respondent’s jurisdiction, in which a higher score indicates greater perceived benefit.

### Inner context determinants of tax implementation

Measures in this domain will characterize organizational and individual-level factors that might influence real and perceived implementation outcomes. *Implementation climate* related to the tax will be assessed using an adapted version of the Educational Support for Evidence-based Practice sub-scale (*α* = 0.84) of the Implementation Climate Scale [97]. These items will be used to create a mean score. Respondents’ perceptions of *their organization’s role in the tax implementation* will be assessed by asking them to identify one of three roles, each of which corresponds with one of the three actor types in Leeman et al.’s typology (i.e., delivery system actors, support system actors, synthesis and translation system actors). Respondents’ *individual roles in tax implementation processes* will be assessed by seven items that assess the extent of involvement in different activities related to tax implementation. If internal consistency is reasonable (i.e., *α* ≥ 0.70), we will calculate the mean of these scores to create an aggregate measure of involvement in tax implementation, in which a higher score indicates greater involvement.

### Outer context determinants of tax implementation

Measures in this domain will characterize perceptions of external factors that could have real and perceived impact on implementation outcomes of the earmarked tax. The selection of constructs informed by a review of outer-context measures in mental health implementation research [71]. *Cosmopolitanism* will be measured by assessing the frequency inter-organization collaboration between the respondent and six external organizations on issues related to implementation of the earmarked tax. These items have been used in research with county mental health agency officials, where they demonstrated high internal consistency (*α* = 0.84) and were strongly and independently associated with the frequency of using research evidence in policy implementation. We will calculate the mean score across these items to create an aggregate score. *Peer pressure* will be measured by five items which assess the extent to which respondents perceive five groups (e.g., the general public, policymakers, consumers of services) as strongly supporting the earmarked tax. This measure is conceptualized as an indicator of the sociopolitical context in which policy implementation occurs [98, 99].

### Innovation determinants

Drawing from the concept of *attributes of innovations* in Rogers’ theory of the Diffusion of Innovations, the survey will assess perceptions of the earmarked tax across the five dimensions: complexity, observability, trialability, compatibility, and relative advantage. These constructs have been assessed in prior mental health policy implementation research [100]. Each dimension will be assessed by two items. If internal consistency is reasonable (i.e., *α* ≥ 0.70), we will sum items within each dimension to create attribute of innovation sub-scales and also sum responses across all items to create an aggregate measure of perceptions of the attributes of the tax. Items will be coded so that a higher score equates to more favorable perceptions of the attributes of the tax.

### Acceptability and feasibility of policy implementation strategies

Measures in this domain will assess attitudes towards the acceptability and feasibility of respondents using different types of implementation strategies to increase the reach of EBPs with tax revenue. These constructs will be assessed by Weiner et al.’s measures of acceptability (four items, *α* = 0.85) and feasibility (four times, *α* = 0.89) [101]. Respondents will rate the acceptability and feasibility of using the five types of implementation strategies proposed in Leeman et al.’s implementation strategy taxonomy (Dissemination strategies, Implementation process strategies, Integration strategies, Capacity-building strategies, and Scale-up strategies) [11]. Each type of strategy will be concisely defined in the survey in relation to the earmarked tax policy. We will sum responses to calculate aggregate acceptability and feasibility scores for each type of implementation strategy.

### Analysis of survey data

There will be at least two primary sets of analyses. In one set of analyses the dependent variable will be perceptions of the impacts of the earmarked tax. The independent variables will be inner context, outer context, and innovation variables. Multivariate regression models will produce adjusted estimates of associations between these constructs and perceived impacts of the tax. These analyses will identify potential targets for implementation strategies (e.g., improve implementation climate related to the tax) and tax design (e.g., refine/develop taxes with attributes that are perceived more favorable) that could enhance the perceived and actual benefits of earmarked taxes for mental health services.

In the other set of analyses, the dependent variables will be perceptions of the acceptability and feasibility of each type of implementation strategy and independent variables will be inner context, outer context, and innovation variables. These analyses will shed light on the types of strategies that could be most readably deployed to improve the reach of EBPs with earmarked tax revenue in different contexts. Both sets of analyses will assess heterogeneity in the direction and magnitude of associations by respondent role/level of involvement in tax implementation and the “actor type” of their organization.

### Interview respondents, recruitment, and qualitative data collection

Interviews will be conducted in eight or more tax implementing counties, at least four in California and four in Washington. We focus on these two states because both were passed in 2005 but vary dramatically in tax design [6]. Interviews may also be conducted in additional states based on aim 1 legal mapping findings. Counties will be selected in consultation with practice partners considering factors such as county population size and rural/urbanicity. The survey contact database will be used to identify potential interview respondents, as well as a snowballing recruitment strategy in which interview respondents will be asked if there are other individuals in their county we should interview about tax implementation. Approximately 50 interviews will be conducted. This number should allow for thematic saturation across about four strata of interview respondents [102], varying across attributes such as tax design and rural/urban setting.

### Interview guide development and analysis

A semi-structured interview guide will include 7–9 open-ended questions, each with multiple probing questions (interview guide as Supplemental File B). Interview questions will focus on furthering understanding perceptions of attributes of the tax, how decisions are made about which services and programs to fund with tax revenue, and general attitudes about earmarked taxes as a mental health financing strategy in the USA. Interview data will primarily be analyzed using the framework development process detailed below.

### Aim 3 methods

Aim 3 methods involve a systematic framework development process [15] to integrate quantitative and qualitative data. The overarching *objective of this process is to develop a conceptual policy implementation framework* to improve tax design and guide the selection implementation strategies that can help ensure that earmarked taxes increase the reach of EBPs in community behavioral health settings. According to Nilsen’s typology of D&I frameworks [103], the product will be both a *determinants framework*, as it will depict barriers and facilitators to earmarked tax dollars increasing access to EBPs, and a *process framework*, as it will provide concrete guidance about specific implementation strategies that might be well-suited for different contexts.

### Analysis of interview data, integration with survey data, and framework development

Framework development will be guided by Jabareen’s six step process for framework development [15].

1. *Review quantitative findings and relevant frameworks.* This step serves to identify, a priori, concepts that have potential utility in the framework. Key findings from the quantitative survey will be transformed into preliminary concepts (e.g., association between perceptions of the flexibility of tax spending and perceived benefits of the tax) with names and definitions. Existing policy D&I frameworks, such as recent advances in integration of policy into the EPIS framework [82], policy implementation frameworks from fields public administration and management research and political science, and other scholarship on policy implementation will be used to identify potentially important concepts.
2. *Read, code, and categorize interview data*. This step consists of organizing interview data into categories at a low level of abstraction. Transcripts will be read by two coders who will assign sections of text to inductively generated categories and create category names and definitions.
3. *Establish core concepts.* This step entails coding transcripts at a higher level of abstraction and creating core concepts that reflect commonalities between multiple categories. Concepts will be created through an iterative process using analytic techniques such as coding matrices, quote tables, and searching for divergent findings.
4. *Create framework.* The purpose of this step is to synthesize quantitative and qualitative findings and create a conceptual framework that provides a comprehensive understanding of barriers and facilitators to earmarked tax dollars increasing reach of EBPs, offering concrete guidance about specific implementation strategies that might work well in different contexts. To achieve this, a diagram will be created that depicts sequences, and inter-relationships among concepts related to inner context, outer context, and innovation determinants.

### Framework dissemination

A two-page summary of the framework, complete with recommendations for tax design and implementation strategies that are perceived as acceptable and feasible, will be created. Findings from policymaker-focused dissemination research will inform decisions about the content of the summary and channels through which it is distributed [16–21]. The summary will be tailored for jurisdictions and e-mailed to policymakers and implementers (e.g., state legislators, oversite officials, local mental health agency leaders) as well as intermediary organizations.

## Discussion

The study may encounter a series of logistical challenges. In aim 1, potential challenges relate to the fact that local (e.g., county, city, township) policies are not captured as routinely in national legal databases than state policies. For this reason, a wide range of data sources will be searched and interviews with key informants will be leverage to identify all policies that earmark taxes for mental health services. Some districts may not have a retrievable record of the earmarked tax revenue and/or date of enactment. Another aim 1 challenge relates to identifying tax revenue information that is earmarked *specifically for mental health* as opposed to mental health in addition social services, which may be co-funded with earmarked tax revenue. In aim 2, challenges will relate to identifying individuals involved with earmarked tax implementation in each jurisdiction, their up-to-date contact information, and achieving a reasonable response rate. Strategies such as personalizing e-mail communication, conducting telephone follow-up, and working with professional associations to endorse the survey will be used to help achieve a reasonable response rate.

## Supplementary Information

**Additional file 1. **Domains of the web-based survey.

**Additional file 2. **Interview guide.

## Data Availability

The datasets used created by the current study are available from the corresponding author on reasonable request.

## References

[1] Jaramillo ET, Willging CE, Green AE, Gunderson LM, Fettes DL, Aarons GA. “Creative Financing”: funding evidence-based interventions in human service systems. The journal of behavioral health services & research. 2019;46(3):366-383. 10.1007/s11414-018-9644-5. 10.1007/s11414-018-9644-5 PMC6816239 30535899

[2] Bruns EJ, Parker EM, Hensley S, Pullmann MD, Benjamin PH, Lyon AR, et al. The role of the outer setting in implementation: associations between state demographic, fiscal, and policy factors and use of evidence-based treatments in mental healthcare. Implement Sci. 2019;14(1):96. 10.1186/s13012-019-0944-9. 10.1186/s13012-019-0944-9 PMC6854683 31722738

[3] Raghavan R, Bright CL, Shadoin AL (2008). Toward a policy ecology of implementation of evidence-based practices in public mental health settings. Implement Sci.

[4] Dopp AR, Narcisse MR, Mundey P, Silovsky JF, Smith AB, Mandell D, et al. A scoping review of strategies for financing the implementation of evidence-based practices in behavioral health systems: state of the literature and future directions. Implementation Research and Practice. 2020;1:2633489520939980. 10.1177/2633489520939980 PMC9924261 37089129

[5] Powell BJ, Waltz TJ, Chinman MJ, Damschroder LJ, Smith JL, Matthieu MM, et al. A refined compilation of implementation strategies: results from the Expert Recommendations for Implementing Change (ERIC) project. Implement Sci. 2015;10:21. 10.1186/s13012-015-0209-1. 10.1186/s13012-015-0209-1 PMC4328074 25889199

[6] Purtle J, Stadnick NA. Earmarked taxes as a policy strategy to increase funding for behavioral health services. Psychiatr Serv. 2020;71(1):100-104. 10.1176/appi.ps.201900332. 10.1176/appi.ps.201900332 PMC6939131 31590621

[7] Purtle J, Brinson K, Stadnick NA. Earmarking excise taxes on recreational cannabis for investments in mental health: an underused financing strategy. JAMA Health Forum. 2022;3(4):e220292. 10.1001/jamahealthforum.2022.0292. 10.1001/jamahealthforum.2022.0292 36218958

[8] Wilkinson M (1994). Paying for public spending: is there a role for earmarked taxes?. Fisc Stud.

[9] Hoagwood KE, Purtle J, Spandorfer J, Peth-Pierce R, Horwitz SM (2020). Aligning dissemination and implementation science with health policies to improve children’s mental health. Am Psychol.

[10] Aarons GA, Hurlburt M, Horwitz SM (2011). Advancing a conceptual model of evidence-based practice implementation in public service sectors. Adm Policy Ment Health.

[11] Leeman J, Birken SA, Powell BJ, Rohweder C, Shea CM (2017). Beyond “implementation strategies”: classifying the full range of strategies used in implementation science and practice. Implement Sci.

[12] Ramanathan T, Hulkower R, Holbrook J, Penn M. Legal epidemiology: the science of law. Journal of Law, Medicine & Ethics. 2017;45(S1):69-72. 10.1177/1073110517703329 PMC5690565 28661299

[13] Burris S. A technical guide for policy surveillance. Temple University Legal Studies Research Paper. 2014 (2014-34).

[14] Burris S, Wagenaar AC, Swanson J, Ibrahim JK, Wood J, Mello MM (2010). Making the case for laws that improve health: a framework for public health law research. Milbank Q.

[15] Jabareen Y (2009). Building a conceptual framework: philosophy, definitions, and procedure. Int J Qual Methods.

[16] Purtle J, Lê-Scherban F, Nelson KL, Shattuck PT, Proctor EK, Brownson RC (2020). State mental health agency officials’ preferences for and sources of behavioral health research. Psychol Serv.

[17] Purtle J, Lê-Scherban F, Wang X, Shattuck PT, Proctor EK, Brownson RC (2018). Audience segmentation to disseminate behavioral health evidence to legislators: an empirical clustering analysis. Implement Sci.

[18] Purtle J, Brownson RC, Proctor EK (2017). Infusing science into politics and policy: the importance of legislators as an audience in mental health policy dissemination research. Adm Policy Ment Health.

[19] Purtle J, Dodson E, Brownson R. Policy dissemination research. Dissemination and implementation research in health: Translating science to practice. 2018:433–48.

[20] Purtle J, Dodson EA, Brownson RC (2016). Uses of research evidence by State legislators who prioritize behavioral health issues. Psychiatr Serv.

[21] Purtle J, Dodson EA, Nelson K, Meisel ZF, Brownson RC (2018). Legislators’ sources of behavioral Health Research and preferences for dissemination: variations by political party. Psychiatr Serv.

[22] Bös D (2000). Earmarked taxation: welfare versus political support. J Public Econ.

[23] Buchanan JM (1963). The economics of earmarked taxes. J Polit Econ.

[24] Martin IW, Lopez JL, Olsen L (2019). Policy design and the politics of city revenue: evidence from california municipal ballot measures. Urban Affairs Review.

[25] Leland S, Chattopadhyay J, Maestas C, Piatak J (2021). Policy venue preference and relative trust in government in federal systems. Governance.

[26] Pew Research Center. Public trust in government: 1958–2022. https://www.pewresearch.org/politics/2022/06/06/public-trust-in-government-1958-2022/.

[27] Tahk SC. Public Choice Theory and Earmarked Taxes. Tax L Rev. 2014;68:755.

[28] World Health Organization. Health taxes: a primer for WHO staff. World Health Organization; 2018.

[29] Crowley GR, Hoffer AJ. Earmarking Tax Revenues: Leviathan's Secret Weapon? For Your Own Good: Taxes, Paternalism, and Fiscal Discrimination in the Twenty-First Century. Arlington, VA: Mercatus Center at George Mason University; 2018.

[30] Dye RF, McGuire TJ (1992). The effect of earmarked revenues on the level and composition of expenditures. Public Finance Q.

[31] Bell E, Wehde W, Stucky M (2020). Supplement or supplant? Estimating the impact of state lottery earmarks on higher education funding. Educ Finance Policy.

[32] Nguyen-Hoang P (2015). Volatile earmarked revenues and state highway expenditures in the United States. Transportation.

[33] Wright A, Smith KE, Hellowell M (2017). Policy lessons from health taxes: a systematic review of empirical studies. BMC Public Health.

[34] Chaloupka FJ, Powell LM, Warner KE (2019). The use of excise taxes to reduce tobacco, alcohol, and sugary beverage consumption. Annu Rev Public Health.

[35] Forberger S, Reisch L, Meshkovska B, Lobczowska K, Scheller DA, Wendt J, Christianson L, Frense J, Steinacker JM, Luszczynska A, Zeeb H (2022). Sugar-sweetened beverage tax implementation processes: results of a scoping review. Health Res Policy Syst.

[36] Purtle J, Langellier B, Lê-Scherban F (2018). A case study of the Philadelphia sugar-sweetened beverage tax policymaking process: implications for policy development and advocacy. J Public Health Manag Pract.

[37] Jayakumar KL, Lipoff JB (2018). Tax collections and spending as a potential measure of health policy association with indoor tanning, 2011–2016. JAMA Dermatol.

[38] Elder RW, Lawrence B, Ferguson A, Naimi TS, Brewer RD, Chattopadhyay SK, Toomey TL, Fielding JE, Task Force on Community Preventive Services. The effectiveness of tax policy interventions for reducing excessive alcohol consumption and related harms. American journal of preventive medicine. 2010 Feb 1;38(2):217–29. 10.1016/j.amepre.2009.11.005 PMC3735171 20117579

[39] Chaloupka FJ, Yurekli A, Fong GT (2012). Tobacco taxes as a tobacco control strategy. Tob Control.

[40] Frank RG. Better but not well: Mental health policy in the United States since 1950. JHU Press; 2006.

[41] Alegría M, Frank RG, Hansen HB, Sharfstein JM, Shim RS, Tierney M. Transforming Mental Health And Addiction Services: Commentary describes steps to improve outcomes for people with mental illness and addiction in the United States. Health Aff (Millwood). 2021;40(2):226-34. 10.1377/hlthaff.2020.01472. Epub 2021 Jan 21. 10.1377/hlthaff.2020.01472 33476189

[42] Grob GN (1994). Government and mental health policy: a structural analysis. Milbank Q.

[43] U.S. Departments of Labor, Health and Human Services, Treasury. 2022 MHPAEA Report to Congress. https://www.dol.gov/sites/dolgov/files/EBSA/laws-and-regulations/laws/mental-health-parity/report-to-congress-2022-realizing-parity-reducing-stigma-and-raising-awareness.pdf.

[44] Barry CL, Huskamp HA, Goldman HH (2010). A political history of federal mental health and addiction insurance parity. Milbank Q.

[45] California Department of Health Care Services. Behavioral Health Information Notice No: 21–057. Mental Health Services Act (MHSA) Allocation and Methodology for FiscalYear (FY) 2021–22. https://www.dhcs.ca.gov/Documents/CSD_YV/BHIN/BHIN-21-057.pdf.

[46] Thom M (2022). Can additional funding improve mental health outcomes? Evidence from a synthetic control analysis of California’s millionaire tax. PLoS ONE.

[47] Collins RL, Wong EC, Breslau J, Burnam MA, Cefalu M, Roth E (2019). Social marketing of mental health treatment: California’s mental illness stigma reduction campaign. Am J Public Health.

[48] Starks SL, Arns PG, Padwa H, Friedman JR, Marrow J, Meldrum ML, Bromley E, Kelly EL, Brekke JS, Braslow JT (2017). System transformation under the California Mental Health Services Act: implementation of full-service partnerships in LA County. Psychiatr Serv.

[49] Ashwood JS, Kataoka SH, Eberhart NK, Bromley E, Zima BT, Baseman L, Marti FA, Kofner A, Tang L, Azhar GS, Chamberlin M. Evaluation of the mental health services act in Los Angeles County: implementation and outcomes for key programs. Rand Health Quarterly. 2018 Aug;8(1). 10.7249/rhq-08-01-02 PMC6075804 30083423

[50] Brookman-Frazee L, Stadnick N, Roesch S, Regan J, Barnett M, Bando L, Innes-Gomberg D, Lau A (2016). Measuring sustainment of multiple practices fiscally mandated in children’s mental health services. Adm Policy Ment Health.

[51] Bruns EJ, Pullmann MD, Weathers ES, Wirschem ML, Murphy JK (2012). Effects of a multidisciplinary family treatment drug court on child and family outcomes: results of a quasi-experimental study. Child Maltreat.

[52] CNN. 2022. 90% of US adults say the United States is experiencing a mental health crisis, CNN/KFF poll finds. https://www.cnn.com/2022/10/05/health/cnn-kff-mental-health-poll-wellness/index.html#:~:text=90%25%20of%20US%20adults%20say%20the%20United%20States%20is%20experiencing,crisis%2C%20CNN%2FKFF%20poll%20finds&text=An%20overwhelming%20majority%20of%20people,with%20the%20Kaiser%20Family%20Foundation.

[53] McGinty EE, Goldman HH, Pescosolido BA, Barry CL (2018). Communicating about mental illness and violence: balancing stigma and increased support for services. J Health Polit Policy Law.

[54] Stone EM, McGinty EE (2018). Public willingness to pay to improve services for individuals with serious mental illness. Psychiatr Serv.

[55] Johnson FR, Gonzalez JM, YANG JC, Ozdemir S, Kymes S. Who would pay higher taxes for better mental health? Results of a large-sample national choice experiment. The Milbank Quarterly. 2021;99(3):771–93. 10.1111/1468-0009.12523 PMC8452366 34375477

[56] DeLeon P, DeLeon L (2002). What ever happened to policy implementation? An alternative approach. Journal of public administration research and theory.

[57] Nilsen P, Ståhl C, Roback K, Cairney P (2013). Never the twain shall meet?–a comparison of implementation science and policy implementation research. Implement Sci.

[58] Bogenschneider K, Bogenschneider BN (2020). Empirical evidence from state legislators: how, when, and who uses research. Psychol Public Policy Law.

[59] Bardach E (1977). The implementation game: what happens after a bill becomes a law.

[60] Implementation WW, Peters B, Pierre J (2006). Handbook of Public Policy.

[61] Pressman J, Wildavsky A (1984). Implementation: How great expectations in Washington are dashed in Oakland; or why it’s amazing that federal programs work at all expanded.

[62] Purtle J, Peters R, Brownson RC (2015). A review of policy dissemination and implementation research funded by the National Institutes of Health, 2007–2014. Implement Sci.

[63] Emmons KM, Gandelman E (2019). Translating behavioral medicine evidence to public policy. J Behav Med.

[64] Purtle J, Nelson KL, Bruns EJ, Hoagwood KE. Dissemination strategies to accelerate the policy impact of children’s mental health services research. Psychiatric Services. 2020;71(11):1170-8. 10.1176/appi.ps.201900527. Epub 2020 Jun 10. 10.1176/appi.ps.201900527 PMC9721469 32517640

[65] Emmons KM, Chambers DA (2021). Policy implementation science–an unexplored strategy to address social determinants of health. Ethnicity Disease.

[66] Emmons KM, Chambers D, Abazeed A (2021). Embracing policy implementation science to ensure translation of evidence to cancer control policy. Transl Behav Med.

[67] Oh A, Abazeed A, Chambers DA (2021). Policy implementation science to advance population health: the potential for learning health policy systems. Front Public Health.

[68] Brownson RC, Kumanyika SK, Kreuter MW, Haire-Joshu DJIS. Implementation science should give higher priority to health equity. Implement Sci. 2021;16(1):28. 10.1186/s13012-021-01097-0. 10.1186/s13012-021-01097-0 PMC7977499 33740999

[69] Ashcraft LE, Quinn DA, Brownson RC (2020). Strategies for effective dissemination of research to United States policymakers: a systematic review. Implement Science.

[70] Allen P, Pilar M, Walsh-Bailey C, Hooley C, Mazzucca S, Lewis CC, Mettert KD, Dorsey CN, Purtle J, Kepper MM, Baumann AA (2020). Quantitative measures of health policy implementation determinants and outcomes: a systematic review. Implement Science.

[71] McHugh S, Dorsey CN, Mettert K, Purtle J, Bruns E, Lewis CC (2020). Measures of outer setting constructs for implementation research: a systematic review and analysis of psychometric quality. Implement Res Pract.

[72] Pilar M, Jost E, Walsh-Bailey C, Powell BJ, Mazzucca S, Eyler A, Purtle J, Allen P, Brownson RC (2022). Quantitative measures used in empirical evaluations of mental health policy implementation: A systematic review. Implement Res Pract.

[73] Crowley DM, Scott JT, Long EC, Green L, Israel A, Supplee L, et al. Lawmakers’ use of scientific evidence can be improved. Proc Natl Acad Sci USA. 2021;118(9):e2012955118. 10.1073/pnas.2012955118. 10.1073/pnas.2012955118 PMC7936366 33593938

[74] Williamson A, Barker D, Green S, D’Este C, Davies HT, Jorm L (2019). Increasing the capacity of policy agencies to use research findings: a stepped-wedge trial. Health Res Policy Syst.

[75] Long EC, Pugel J, Scott JT, Charlot N, Giray C, Fernandes MA, et al. Rapid-cycle experimentation with state and federal policymakers for optimizing the reach of racial equity research. Am J Public Health. 2021;111(10):1768-71. 10.2105/AJPH.2021.306404. Epub 2021 Sep 9. 10.2105/AJPH.2021.306404 PMC8522578 34499535

[76] Levine AS. Single conversations expand practitioners’ use of research: evidence from a field experiment. PS: Political Science & Politics. 2021;54(3):432-7.

[77] Niederdeppe J, Winett LB, Xu Y, Fowler EF, Gollust SE. Evidence-based message strategies to increase public support for state investment in early childhood education: results from a longitudinal panel experiment. Milbank Q. 2021;99(4):1088-131. 10.1111/1468-0009.12534. Epub 2021 Aug 17. 10.1111/1468-0009.12534 PMC8718583 34402554

[78] Niederdeppe J, Roh S, Dreisbach C (2016). How narrative focus and a statistical map shape health policy support among state legislators. Health Commun.

[79] Winett LB, Niederdeppe J, Xu Y, Gollust SE, Fowler EF. When “Tried and True” advocacy strategies backfire: narrative messages can undermine state legislator support for early childcare policies. The Journal of Public Interest Communications. 2021;5(1):45.

[80] Brownson RC, Dodson EA, Stamatakis KA, Casey CM, Elliott MB, Luke DA (2011). Communicating evidence-based information on cancer prevention to state-level policy makers. J Natl Cancer Inst.

[81] Bullock HL, Lavis JN, Wilson MG, Mulvale G, Miatello A (2021). Understanding the implementation of evidence-informed policies and practices from a policy perspective: a critical interpretive synthesis. Implement Sci.

[82] Crable EL, Lengnick-Hall R, Stadnick NA, Moullin JC, Aarons GA (2022). Where is “policy” in dissemination and implementation science? Recommendations to advance theories, models, and frameworks: EPIS as a case example. Implement Sci.

[83] Purtle J, Borchers B, Clement T, Mauri A (2018). Inter-agency strategies used by state mental health agencies to assist with federal behavioral health parity implementation. J Behav Health Serv Res.

[84] Crable EL, Benintendi A, Jones DK, Walley AY, Hicks JM, Drainoni ML (2022). Translating Medicaid policy into practice: policy implementation strategies from three US states’ experiences enhancing substance use disorder treatment. Implement Sci.

[85] Olson JR, Azman A, Estep KM, Coviello KA, Robshaw S, Bruns EJ (2021). Influences of inner and outer settings on wraparound implementation outcomes. Glob Implement Res Appl.

[86] Bruns EJ, Parker EM, Hensley S, Pullmann MD, Benjamin PH, Lyon AR, Hoagwood KE (2019). The role of the outer setting in implementation: associations between state demographic, fiscal, and policy factors and use of evidence-based treatments in mental healthcare. Implement Sci.

[87] McGinty EE, Tormohlen KN, Barry CL, Bicket MC, Rutkow L, Stuart EA (2021). Protocol: mixed-methods study of how implementation of US state medical cannabis laws affects treatment of chronic non-cancer pain and adverse opioid outcomes. Implement Sci.

[88] Dopp AR, Hunter SB, Godley MD, Pham C, Han B, Smart R, Cantor J, Kilmer B, Hindmarch G, González I, Passetti LL (2022). Comparing two federal financing strategies on penetration and sustainment of the adolescent community reinforcement approach for substance use disorders: protocol for a mixed-method study. Implement Sci Commun.

[89] Purtle J, Lê-Scherban F, Shattuck P, Proctor EK, Brownson RC (2017). An audience research study to disseminate evidence about comprehensive state mental health parity legislation to US State policymakers: protocol. Implement Sci.

[90] Crable EL, Grogan CM, Purtle J, Roesch SC, Aarons GA (2023). Tailoring dissemination strategies to increase evidence-informed policymaking for opioid use disorder treatment: study protocol. Implement Sci Commun.

[91] Moullin JC, Dickson KS, Stadnick NA, Rabin B, Aarons GA (2019). Systematic review of the exploration, preparation, implementation, sustainment (EPIS) framework. Implement Sci.

[92] Rogers EM, Singhal A, Quinlan MM (2014). Diffusion of innovations.

[93] Hsieh H-F, Shannon SE (2005). Three approaches to qualitative content analysis. Qual Health Res.

[94] Purtle J, Nelson KL, Lengnick-Hall R, Horwitz SM, Palinkas LA, McKay MM, Hoagwood KE (2022). Inter-agency collaboration is associated with increased frequency of research use in children’s mental health policy making. Health Serv Res.

[95] Purtle J, Nelson KL, Horwitz SM, McKay MM, Hoagwood KE (2021). Determinants of using children’s mental health research in policymaking: variation by type of research use and phase of policy process. Implement Sci.

[96] Purtle J, Nelson KL, Horwitz SM, Palinkas LA, McKay MM, Hoagwood KE (2022). Impacts of COVID-19 on mental health safety net services for youths: a national survey of agency officials. Psychiatr Serv.

[97] Ehrhart MG, Aarons GA, Farahnak LR (2014). Assessing the organizational context for EBP implementation: the development and validity testing of the Implementation Climate Scale (ICS). Implement Sci.

[98] Purtle J (2020). Public opinion about evidence-informed health policy development in US Congress. Transl Behav Med.

[99] Nilsen P, Bernhardsson S (2019). Context matters in implementation science: a scoping review of determinant frameworks that describe contextual determinants for implementation outcomes. BMC Health Serv Res.

[100] Stewart RE, Marcus SC, Hadley TR, Hepburn BM, Mandell DS (2018). State adoption of incentives to promote evidence-based practices in behavioral health systems. Psychiatr Serv.

[101] Weiner BJ, Lewis CC, Stanick C, Powell BJ, Dorsey CN, Clary AS, Boynton MH, Halko H (2017). Psychometric assessment of three newly developed implementation outcome measures. Implement Sci.

[102] Hennink M, Kaiser BN (2022). Sample sizes for saturation in qualitative research: a systematic review of empirical tests. Soc Sci Med.

[103] Nilsen P (2015). Making sense of implementation theories, models and frameworks. Implement Sci.

