# Supplementary material for: A policy implementation study of earmarked taxes for mental health services: study protocol
Source: Implement Sci Commun. 2023 Mar 31;4:37. doi: 10.1186/s43058-023-00408-4 (PMC10067193; doi:10.1186/s43058-023-00408-4)
Supplement: Supplementary file 1 — Additional file 1. Domains of the web-based survey. [file 43058_2023_408_MOESM1_ESM.docx]

FINAL MH Tax Survey 9.16.22

Start of Block: Consent

Q1 **Survey Consent Form for IRB-FY2022-6337**

You have been invited to take part in a research study to learn more about local public and community organization officials’ experiences implementing earmarked taxes for behavioral health, decision-making processes related to tax-funded programs, and services and perceptions of the acceptability and feasibility of different types of implementation strategies. This study is being conducted by Dr. Jonathan Purtle, College of Global Public Health, New York University and is funded by the National Institute of Mental Health.

If you agree to be in this study, you will be asked to do the following:
• Answer questions about your experiences implementing earmarked taxes for behavioral health, decision-making processes related to tax-funded programs and services and perceptions of the acceptability and feasibility of different types of implementation strategies.

The survey will take about 15 minutes to complete. There are no known risks associated with your participation in this research beyond those of everyday life.

Although you will receive no direct benefits, this research may help the investigator identify strategies to help support and improve the implementation of earmarked taxes for behavioral health services. As a thank you for completing the survey, we will provide you with a $20 gift card via email after completion.

Confidentiality of your research records will be strictly maintained. Your survey responses will be collected in a dataset that identifies you according to a randomly assigned code number. Personal identifiers such as your name or agency will not be contained in the survey dataset with your responses. This research is covered by a Certificate of Confidentiality from the National Institutes of Health. Researchers with this Certificate will not disclose or use information that may identify you in any federal, state, or local civil, criminal, administrative, legislative, or other action, suit, or proceeding, even if there is a court subpoena. Exceptions include:
• A federal, state, or local law requires disclosure, such as Your explicit approval for the researchers to release your name and/or personally identifiable information.

Participation in this study is voluntary. You may refuse to participate or stop taking the survey at any time without penalty. You have the right to skip or not answer any questions you prefer not to answer.

If there is anything about the study or your participation that is unclear or that you do not understand, if you have questions or wish to report a research-related problem, you may contact Jonathan Purtle at 267-546-7541, jonathan.purtle@nyu.edu, 708 Broadway, Room 715 New York, NY, 10003.

For questions about your rights as a research participant, you may contact the University Committee on Activities Involving Human Subjects (UCAIHS), New York University, 665 Broadway, Suite 804, New York, New York, 10012, at ask.humansubjects@nyu.edu or (212) 998-4808. Please reference the study # (IRB-FY2022-6337) when contacting the IRB (UCAIHS).

Click to the next screen if you agree to participate.

| Page Break |  |
| --- | --- |

Q29 Please indicate your state

- California (1)
- Washington (2)
- Missouri (3)
- Illinois (5)
- Colorado (6)

End of Block: Consent

Start of Block: Inner Context: Organizational and individual role in tax implementation

Q2 *First are a couple of questions about your role in the implementation of the earmarked tax for behavioral health (i.e., mental health/substance use) in your jurisdiction.*

| 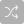 |
| --- |

Q6 Please rate the extent to which have **you have personally** been involved with the following activities related to the earmarked tax for behavioral health in your jurisdiction.

|  | **1= Not involved at all** | **Very involved= 7** |
| --- | --- | --- |

|  | 1 | 2 | 3 | 4 | 5 | 6 | 7 |
| --- | --- | --- | --- | --- | --- | --- | --- |

| Collecting and reporting information for compliance purposes () | 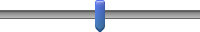 |
| --- | --- |
| Directly providing tax funded services in the community () | 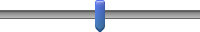 |
| Making decisions about what services to fund with tax revenue () | 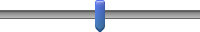 |
| Evaluating the impacts of tax funded services () | 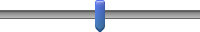 |
| Establishing relationships with external partners related to the tax () | 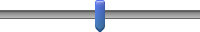 |
| Strategically planning how tax revenue can most optimally be spent () | 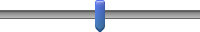 |
| Monitoring how tax revenue is spent for compliance purposes () | 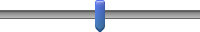 |

End of Block: Inner Context: Organizational and individual role in tax implementation

Start of Block: Perceptions of the benefits and attributes of the tax

Q8 *Next are some questions about your perceptions of the earmarked tax for behavioral health in your jurisdiction.*

| 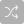 |
| --- |

Q7 Rate the extent to which you agree with the following statements about the **impacts** of the earmarked tax for behavioral health in your jurisdiction.

|  | **1= Strongly disagree** | **Strongly agree= 7** | Not Applicable |
| --- | --- | --- | --- |

|  | 1 | 2 | 3 | 4 | 5 | 6 | 7 |
| --- | --- | --- | --- | --- | --- | --- | --- |

| The tax increases funding for direct behavioral health/social services () | 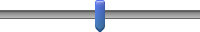 |
| --- | --- |
| The tax increases funding for improvements to behavioral health/social services systems () | 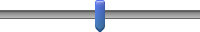 |
| The tax increases the number of people served by evidence-based practices () | 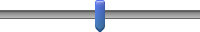 |
| The tax increases flexibility to address complex behavioral health/social serviced needs () | 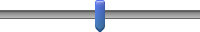 |
| The tax increases access to direct behavioral health/social services for people with the highest need () | 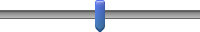 |
| The tax is increases public awareness about behavioral health issues () | 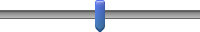 |
| The tax decreases stigma about behavioral health issues () | 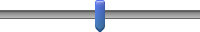 |
| The tax decreases funding from other sources (e.g., general county/state budgets) for behavioral health/social services () | 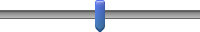 |
| The tax increases transparency about behavioral health/social services systems () | 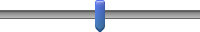 |
| The tax increases unjustified public/political scrutiny about behavioral health/social services systems () | 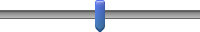 |

| 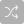 |
| --- |

Q26 Rate the extent to which agree with the following statements about the **characteristics** of the earmarked tax for behavioral health in your jurisdiction.

|  | **1= Strongly disagree** | **Strongly agree= 7** | Not Applicable |
| --- | --- | --- | --- |

|  | 1 | 2 | 3 | 4 | 5 | 6 | 7 |
| --- | --- | --- | --- | --- | --- | --- | --- |

| It is hard to understand what is and is not a permissible use of revenue from the tax () | 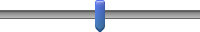 |
| --- | --- |
| It is complicated to satisfy reporting requirements related to using of revenue from the tax () | 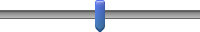 |
| The impact of the tax on the number of people who receive services is easy to observe () | 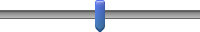 |
| The impact of the tax on the behavioral health status of communities is easy to observe () | 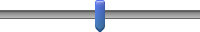 |
| The rules related to how revenue from the tax can be spent can be easily changed to address emergent needs () | 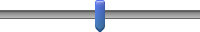 |
| The tax allows behavioral health service organizations to try new services assess whether they meet needs before taking the services to scale () | 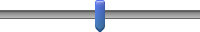 |
| The tax is flexible enough to allow behavioral health service organizations to meet the unique needs of the communities they serve () | 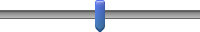 |
| The tax is compatible with the financing structures of behavioral health service organizations () | 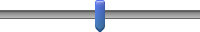 |
| It is better to have the tax than not () | 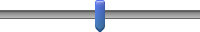 |
| The tax is better than alternative strategies to increase funding for behavioral health services () | 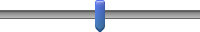 |

End of Block: Perceptions of the benefits and attributes of the tax

Start of Block: Outer context: Cosmopolitanism and Peer-pressure

Q10 *Next is a question about your perceptions of support for the earmarked tax for behavioral health in your jurisdiction among different groups.*

| 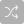 |
| --- |

Q11 Rate the extent to which you agree that there is **strong support for the tax** among….

|  | **1= Completely disagree** | **Completely agree= 7** | Not Applicable |
| --- | --- | --- | --- |

|  | 1 | 2 | 3 | 4 | 5 | 6 | 7 |
| --- | --- | --- | --- | --- | --- | --- | --- |

| The general public in my jurisdiction () | 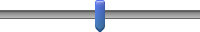 |
| --- | --- |
| Local elected officials in my jurisdiction () | 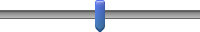 |
| Consumers of behavioral health services in my jurisdiction () | 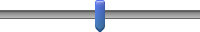 |
| State behavioral health agency officials in my state () | 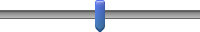 |
| State elected officials in my state () | 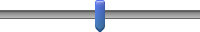 |

Q12 *Next is a question about how often you collaborate with external agencies on issues related to implementation of the earmarked tax.*
**By collaboration we mean the process by which government agencies come together and establish a formal commitment to working together to achieve common goals.**

| 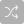 |
| --- |

Q13 Indicate how often you collaborate with each of the following on issues related to implementation of the earmarked tax.

|  | **1= Never** | **Very frequently= 5** | Not Applicable |
| --- | --- | --- | --- |

|  | 1 | 2 | 3 | 4 | 5 |
| --- | --- | --- | --- | --- | --- |

| Local substance use agency/direct service organizations () | 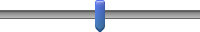 |
| --- | --- |
| Local mental health agency/direct service organizations () | 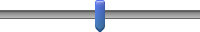 |
| Local public health department/primary care service organizations () | 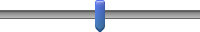 |
| Local education department/schools () | 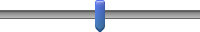 |
| Local child welfare agency/child protective services () | 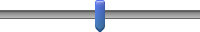 |
| Local justice department/police () | 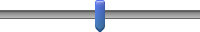 |

End of Block: Outer context: Cosmopolitanism and Peer-pressure

Start of Block: Inner-context, Imp Climate

Q20 *Next is a question about your perceptions of how your organization uses evidence when making decisions about the implementation of the earmarked tax for behavioral health in your jurisdiction.*

| 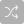 |
| --- |

Q21 Indicate the extent to which you agree with each of the statements below about your organization within the context of decisions about evidence-based practice and implementation of the earmarked tax for behavioral health in your jurisdiction.

|  | **1= Not at all** | **Very great extent= 5** | Not Applicable |
| --- | --- | --- | --- |

|  | 1 | 2 | 3 | 4 | 5 |
| --- | --- | --- | --- | --- | --- |

| One of this organization’s main goals is to use evidence-based practices effectively with earmarked tax revenue () | 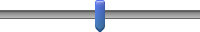 |
| --- | --- |
| People in this organization think that the implementation of evidence-based practices with earmarked tax revenue is important () | 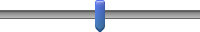 |
| Using evidence-based practices is a top priority in this organization when it comes to making decision about earmarked tax revenue () | 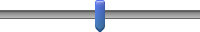 |
| This organization provides conferences, workshops, or seminars focusing on using earmarked tax revenue for evidence-based practices () | 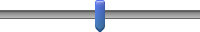 |
| This organization uses earmarked tax revenue to provide evidence-based practice trainings or in-services () | 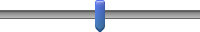 |
| This organization uses earmarked tax revenue to provide evidence-based practice training materials, journals () | 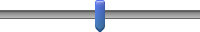 |
| Clinicians in this organization who use evidence-based practices are seen as clinical experts () | 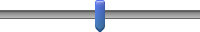 |

End of Block: Inner-context, Imp Climate

Start of Block: Strategies

Q14 *Last are questions about your perceptions of five different types of implementation strategies that your organization could use to maximize the  benefits of the earmarked tax for behavioral health in your jurisdiction.*

Q15 **Dissemination strategies:** These strategies entail your organization **communicating information** to behavioral health service organizations to increase leaders and providers knowledge and improve their attitudes about evidence-based practices that can be funded with earmarked behavioral health tax revenue.

|  | **1= Completely disagree** | **Completely agree= 5** | Not Applicable |
| --- | --- | --- | --- |

|  | 1 | 2 | 3 | 4 | 5 |
| --- | --- | --- | --- | --- | --- |

| Dissemination strategies meet my approval () | 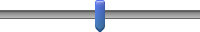 |
| --- | --- |
| Dissemination strategies are appealing to me () | 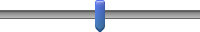 |
| I like dissemination strategies () | 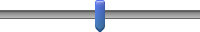 |
| I welcome dissemination strategies () | 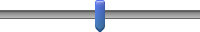 |
| Dissemination strategies seem implementable () | 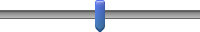 |
| Dissemination strategies seem possible () | 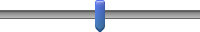 |
| Dissemination strategies seem doable () | 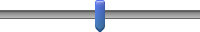 |
| Dissemination strategies seem easy to use () | 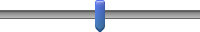 |

Q16 **Implementation process strategies:** These strategies entail your organization **helping behavioral health service organizations’ select** evidence-based practices funded by earmarked behavioral health tax revenue, plan for their integration, and **evaluate** their impacts.

|  | **1= Completely disagree** | **Completely agree= 5** | Not Applicable |
| --- | --- | --- | --- |

|  | 1 | 2 | 3 | 4 | 5 |
| --- | --- | --- | --- | --- | --- |

| Implementation process strategies meet my approval () | 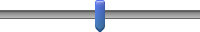 |
| --- | --- |
| Implementation process strategies are appealing to me () | 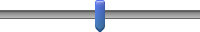 |
| I like implementation process strategies () | 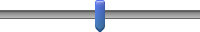 |
| I welcome implementation process strategies () | 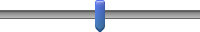 |
| Implementation process strategies seem implementable () | 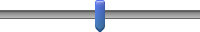 |
| Implementation process strategies seem possible () | 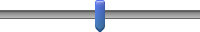 |
| Implementation process strategies seem doable () | 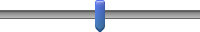 |
| Implementation process strategies seem easy to use () | 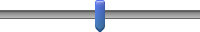 |

Q17  **Integration strategies:** These strategies entail your organization **changing the organizational context** within behavioral health service organizations to ensure the delivery of evidence-based practices funded by earmarked behavioral health tax revenue (e.g., by using clinical reminder systems, quality monitoring activities, and changing professional roles with organizations).

|  | **1= Completely disagree** | **Completely agree= 5** | Not Applicable |
| --- | --- | --- | --- |

|  | 1 | 2 | 3 | 4 | 5 |
| --- | --- | --- | --- | --- | --- |

| Integration strategies meet my approval () | 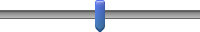 |
| --- | --- |
| Integration strategies are appealing to me () | 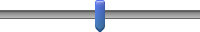 |
| I like integration strategies () | 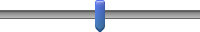 |
| I welcome integration strategies () | 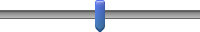 |
| Integration strategies seem implementable () | 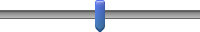 |
| Integration strategies seem possible () | 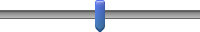 |
| Integration strategies seem doable () | 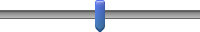 |
| Integration strategies seem easy to use () | 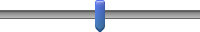 |

Q18  **Capacity-building strategies**: These strategies entail your organization **increasing the capacity** of behavioral health service organizations to select and integrate evidence-based practices funded by earmarked behavioral health tax revenue and evaluate their impacts (e.g., by enhancing the motivation and self-efficacy of leadership and direct service providers).

|  | **1= Completely disagree** | **Completely agree= 5** | Not Applicable |
| --- | --- | --- | --- |

|  | 1 | 2 | 3 | 4 | 5 |
| --- | --- | --- | --- | --- | --- |

| Capacity-building strategies meet my approval () | 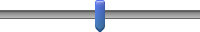 |
| --- | --- |
| Capacity-building strategies are appealing to me () | 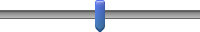 |
| I like capacity-building strategies () | 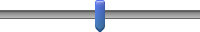 |
| I welcome capacity-building strategies () | 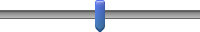 |
| Capacity-building strategies seem implementable () | 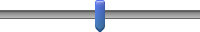 |
| Capacity-building strategies seem possible () | 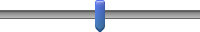 |
| Capacity-building strategies seem doable () | 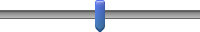 |
| Capacity-building strategies seem easy to use () | 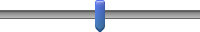 |

Q19  **Scale-up strategies:** These strategies entail your organization **increasing the ability** of behavioral health service organizations to ensure that evidence-based practices funded by earmarked behavioral health tax revenue achieve desired outcomes (e.g., by providing training on evidence-based practice to direct service providers).

|  | **1= Completely disagree** | **Completely agree= 5** | Not Applicable |
| --- | --- | --- | --- |

|  | 1 | 2 | 3 | 4 | 5 |
| --- | --- | --- | --- | --- | --- |

| Scale-up strategies meet my approval () | 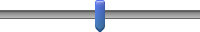 |
| --- | --- |
| Scale-up strategies are appealing to me () | 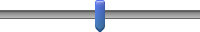 |
| I like scale-up strategies () | 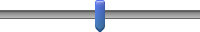 |
| I welcome scale-up strategies () | 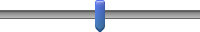 |
| Scale-up strategies seem implementable () | 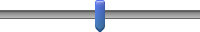 |
| Scale-up strategies seem possible () | 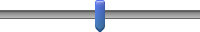 |
| Scale-up strategies seem doable () | 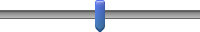 |
| Scale-up strategies seem easy to use () | 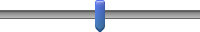 |

End of Block: Strategies

Start of Block: Demographics

Q28 *Final are a few demographic questions.*

| 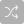 |
| --- |

Q3 Please indicate which categories most accurately describe **your organization’s** role within the context of implementing the earmarked tax for behavioral health in your jurisdiction. Select all that apply.

- Providing direct behavioral health and social services with tax revenue (1)
- Supporting system and capacity building efforts for organizations that provide direct behavioral health and social services with tax revenue (2)
- Reviewing evidence about promising approaches to using earmarked tax revenue and communicating this information to organizations that provide direct behavioral health and social services (3)

Q23 What is your gender?

- Female (1)
- Male (2)
- Non-binary (3)

Q24 What is your race/ethnicity (select all that apply)?

- Black or African American (1)
- White, Non-Hispanic (2)
- Hispanic (3)
- Native American/Alaskan Native (4)
- Asian (5)

Q25 In total, how many years have you worked at your organization?

- Less than one year (1)
- One to two years (2)
- Three to five years (3)
- Six to nine years (4)
- Ten or more years (5)

Q26 What is the highest level of education that you have completed?

- High school or GED (1)
- Some college (2)
- College degree (3)
- Master’s degree (e.g., MS, MA, MPH) (4)
- Doctoral degree (e.g., MD, PhD, JD) (5)

End of Block: Demographics

Start of Block: Incentive and snowball

Q30 Is there anyone else in your jurisdiction that you think we should send this survey to? If so, please enter their name and e-mail address below.

- Name, e-mail 1 (1) __________________________________________________
- Name, e-mail 2 (2) __________________________________________________
- Name, e-mail 3 (3) __________________________________________________
- Name, e-mail 4 (4) __________________________________________________
- Name, e-mail 5 (5) __________________________________________________
- Name, e-mail 6 (6) __________________________________________________
- Name, e-mail 7 (7) __________________________________________________

Q30 If you would like us to follow up with you for an interview about the earmarked tax for behavioral health in your jurisdiction, please enter your e-mail address below.

________________________________________________________________

Q29 We can provide you with a $20 gift card for your completing the survey. We will send you an e-mail with details about how to obtain the gift card. Please enter your email address below. Thank you for completing the survey!

________________________________________________________________

End of Block: Incentive and snowball
