# Supplementary material for: A policy implementation study of earmarked taxes for mental health services: study protocol
Source: Implement Sci Commun. 2023 Mar 31;4:37. doi: 10.1186/s43058-023-00408-4 (PMC10067193; doi:10.1186/s43058-023-00408-4)
Supplement: Supplementary file 2 — Additional file 2. Interview guide. [file 43058_2023_408_MOESM2_ESM.docx]

**Earmarked Tax Interview Guide: Final 11.1.22**

- *Thank you for taking the time to talk with us/me.*
- *I’d like to give you a little bit of background information about the project before we start. As you may know, we recently conducted a national survey of over 200 people involved with the planning and implementation of policies that earmark taxes for behavioral health (i.e., mental health and substance use) services.*
- *The purpose of the interviews we’re conducting now is to explore some of the survey findings in greater depth and explore some things that we didn’t ask about in the survey. The interview questions span three main domains:*
  - *Your perceptions of rules related to the tax and the amount of revenue it generates*
  - *Your perceptions of factors that influence decisions about how earmarked tax revenue is spent*
  - *Your general perceptions of pros and cons of the tax in your jurisdiction*
- *The interview will take about 30 minutes.*
- *We plan to analyze the interviews and share the findings in published peer-review journals and/or research conferences; we will also send you a summary of the research findings.*
- *The interview is confidential, meaning we won’t identify you or your county/city when reporting the results.*
- *With your permission I would like to record the interview so that we can have it transcribed and analyze the results. Is that OK?*

1. To start off, tell me what is your role related to administering the earmarked tax for behavioral health services?

*Next are two broad questions about the pros and cons of the earmarked tax for behavioral health services in* ***[insert name of respondent’s jurisdiction].***

1. In general, what do you see as the top three benefits of the tax?
2. On the other side of the coin, what do you see as up to three major drawbacks, or downsides, of the tax?

*Now I’ll ask a few questions about your perceptions of the policy which created the earmarked tax for behavioral health services in* ***[insert name of respondent’s jurisdiction].***

1. First is a question about the amount of money that the tax generates. Relative to other sources of funding for behavioral health services (e.g., state budget funding, SAMHSA funding) in your jurisdiction, is the amount of revenue from the tax meaningfully large? In other words, is the tax a pretty major funding source or more of a “drop in a bucket?” Tell me a little bit about why or why not.
2. We’ve heard varying perspectives about the flexibility of how tax revenue can be spent. What has been your experience in  **[insert name of respondent’s jurisdiction]**? Tell me a bit about the extent to which you perceive the rules around tax spending as being flexible enough to meet the specific needs of the community you serve.
3. We’ve also heard varying perspectives about the reporting requirements for tax spending. Some folks perceive the reporting requirements as being too extensive and resource intensive, while others perceive them as not been extensive or detailed enough. What do you think about the appropriateness of the tax spending reporting requirements in **[insert name of respondent’s jurisdiction]**?
4. *Next are some questions about factors, that might influence decisions about how earmarked tax revenue is spent in* ***[insert name of respondent’s jurisdiction]****. I’m going to name three factors that might influence these decision-making processes. For each factor, tell me a bit about:*
   - *The extent to which you perceive it as influential,*
   - *Why you think it has as much, or as little, influence as it has, and*
   - *An example, if you can think of one, of the factor having a lot of influence on a spending decision.*
   1. Considerations related to the extent to which the funded services are considered to be **“evidence-based” treatments**, meaning treatments that research suggest are effective.
   - Please describe the extent to which you perceive the extent to which the funded services are considered to be “evidence-based” as influential.
   - Why you think it has as [much/little] influence as it has?
   - Could you provide an example of when the extent to which a service was considered to be “evidence-based” influenced a spending decision?
   1. Considerations related to the **reducing inequities** in access to behavioral health services, defining “inequity” however *you* define it
   - Please describe the extent to which you perceive reducing inequities in access to behavioral health services as influential.
   - Why you think it has as [much/little] influence as it has?
   - Could you provide an example of when reducing inequities in access to behavioral health services influenced a spending decision?
   1. Considerations related to funding services and activities that cannot be funded by other federal, state, and local funding sources
   - Please describe the extent to which you perceive this factor influential.
   - Why you think it has as [much/little] influence as it has?
   - Could you provide an example of when this factor influenced a spending decision?
   1. What other factors influence how the taxes are spent?
   - Please describe the extent to which you perceive this factor influential.
   - Why you think it has as [much/little] influence as it has?
   - Could you provide an example of when this factor influenced a spending decision?
5. OK, final question. An increasing number of U.S. jurisdictions are passing, or considering passing, earmarked taxes for behavioral health services. Based on your experiences in **[insert name of respondent’s jurisdiction]**, what recommendations do you have for folks that are in the early stages of tax design and/or implementation?

*Thanks so much.*

1. We can offer you a $20 Amazon gift card for participating in the interview. Would you like us to send it to you? If so, let me know the e-mail address to send it to.

*Final are three demographic questions that NIH request that we ask.*

What is gender do you identify with (read all response options)?

- Female
- Male
- Non-binary
- Prefer not to answer

Do you identify as Hispanic/Latino (read all response options)?

- No
- Yes
- Prefer not to answer

What is your racial category do you identify with (read all response options)?

- Asian
- Black or African American
- Native American/Alaskan Native
- White
- Prefer not to answer
